# Pharmacokinetics of ceftriaxone, gentamicin, meropenem and vancomycin in liver cirrhosis: a systematic review

**DOI:** 10.1093/jac/dkae310

**Published:** 2024-09-18

**Authors:** M H Comce, R A Weersink, U Beuers, R M van Hest, M A Lantinga

**Affiliations:** Department of Gastroenterology and Hepatology, Amsterdam UMC, University of Amsterdam, Amsterdam Gastroenterology Endocrinology Metabolism, Amsterdam, The Netherlands; Department of Clinical Pharmacy, Deventer Hospital, Deventer, The Netherlands; Department of Gastroenterology and Hepatology, Amsterdam UMC, University of Amsterdam, Amsterdam Gastroenterology Endocrinology Metabolism, Amsterdam, The Netherlands; Department of Pharmacy and Clinical Pharmacology, Amsterdam UMC, University of Amsterdam, Amsterdam Infection & Immunity, Amsterdam Gastroenterology Endocrinology Metabolism, Amsterdam, The Netherlands; Department of Gastroenterology and Hepatology, Amsterdam UMC, University of Amsterdam, Amsterdam Gastroenterology Endocrinology Metabolism, Amsterdam, The Netherlands; European Reference Network on Hepatological Diseases (ERN RARE-LIVER), Hamburg, Germany

## Abstract

**Objectives:**

Patients with liver cirrhosis are prone to develop severe bacterial infections. Pharmacokinetics (PK) of antibiotics in cirrhosis are potentially affected by impaired biotransformation phases 0–3 and consequences of portal hypertension such as portovenous shunting, ascites formation and/or acute kidney injury (AKI). We aimed to elucidate to what extent PK of selected antibiotics and, therefore, dosage recommendations are affected in adults with cirrhosis.

**Methods:**

We performed a systematic search in PubMed, Embase, Cochrane and CINAHL on effects of cirrhosis on PK profiles of ceftriaxone, fosfomycin, gentamicin, meropenem, nitrofurantoin, piperacillin/tazobactam and vancomycin in adults. Antibiotics were selected based on the lack of specific dosing recommendations for adults with cirrhosis. We included studies reporting on ≥1 of the following PK parameters: AUC, half-life (*t*_½_), CL, volume of distribution (*V*_d_), peak (*C*_max_) or trough concentrations (*C*_min_).

**Results:**

We identified 15 studies (ceftriaxone, *n* = 5; gentamicin, *n* = 3; meropenem *n* = 5; vancomycin, *n* = 2), including 379 patients with cirrhosis, of which two were of high quality. No eligible studies were identified for fosfomycin, nitrofurantoin or piperacillin/tazobactam. Ceftriaxone unbound concentration increased in cirrhosis, but was mitigated by increased renal CL. Gentamicin levels in ascitic fluid were comparable to those in plasma. Meropenem PK parameters were not altered in cirrhosis without AKI, but in the presence of AKI a decrease in CL was observed. In contrast, vancomycin CL decreased in advanced cirrhosis.

**Conclusions:**

Available data in studies of mostly moderate quality suggest that PK of ceftriaxone, meropenem and vancomycin are altered in cirrhosis. More advanced PK studies are needed to provide specific dosing recommendations.

## Introduction

Cirrhosis is defined as the end stage of progressive conversion of normal liver architecture into fibrotic liver tissue.^1^ Ongoing liver injury in these patients will lead to the development of clinically relevant portal hypertension. These patients are prone to develop decompensating events with overt clinical signs and symptoms such as ascites, variceal haemorrhage and hepatic encephalopathy.^2^ Of all hospitalized patients with cirrhosis, up to 60% present with an infection or develop one during admission.^3,4^ Compared with patients without cirrhosis, bacterial infections in patients with cirrhosis are associated with a prolonged length of hospital stay and these patients are four times more likely to die with a median 1 month mortality of up to 38%.^5,6^

Successful management of these bacterial infections requires optimal selection and dosing of antibiotic treatment. This is complicated by several pathological alterations due to cirrhosis and portal hypertension that may impact drug absorption, distribution, metabolism and elimination.^7,8^ Specifically, increased intestinal permeability and portosystemic shunts can alter the extent of drug absorption or decrease the first-pass effect, resulting in higher bioavailability. Decreased levels of albumin, accumulation of bilirubin and fluid retention (ascites, oedema) can impact drug distribution, by reducing plasma protein binding, and increase volume of distribution. Diminished activity of drug-metabolizing enzymes due to hepatocellular damage can lead to decreased clearance of drugs undergoing hepatocellular biotransformation and biliary secretion. Finally, bile flow obstruction and acute kidney injury (AKI), the latter reported in up to 50% of hospitalized cirrhotic patients, result in a reduced biliary excretion of drugs and impaired renal clearance, respectively.^9–11^ Adding to the prevalence of AKI, patients with cirrhosis and ascites are prone to develop hepatorenal syndrome (AKI-HRS), a form of prerenal AKI caused by renal vasoconstriction.^12^ The combination of these pathophysiological changes is likely to result in alterations of pharmacokinetic (PK) parameters with the risk of underexposure or overexposure when standard doses of antibiotics are administered.

Special care should go into the selection and dosing of antibiotics in patients with cirrhosis, as suboptimal treatment of the infection (underexposure) or drug toxicity (overexposure) can lead to a detrimental outcome. However, there seems to be a paucity of data on this topic. There is an unmet need regarding to what extent the exposure of antibiotics is affected by a decrease in liver function and portal hypertension, with or without the concomitant presence of AKI. At this time, advice on drug dosing in patients with cirrhosis is absent for most antibiotics.^13^ The limited number of studies that are available in patients with cirrhosis included ill-defined study populations or used outdated techniques to measure antibiotic levels.^14^ Moreover, a systematic overview on the PK changes of frequently prescribed antibiotics in cirrhosis is lacking, as previous studies solely focused on antibiotics that primarily undergo hepatobiliary elimination.^14^

We conducted a systematic review to evaluate the extent and quality of available literature concerning PK alterations of antibiotics in patients with cirrhosis for which currently no evidence-based dosing recommendations exist.

## Materials and methods

### Selection of antibiotics

We selected antibiotics that are commonly used in Dutch clinical practice for the treatment of bacterial infections in patients with cirrhosis or are used as antibiotic prophylaxis when patients with cirrhosis present with acute variceal bleeding. Next, we cross-referenced if no specific dosing advice was available in commonly used drug dosing formularies in the Netherlands with the help of a hospital pharmacist and clinical microbiologist.^15–17^ This led to the following selection of antibiotics: ceftriaxone, meropenem, vancomycin, nitrofurantoin, fosfomycin, gentamicin and piperacillin/tazobactam. We decided to search for PK data on these individual drugs rather than the groups they belong to, as individual antibiotics within a group can have different PK properties and have specific applications in patients with cirrhosis.

### Search strategy and study selection

We conducted a systematic literature search in PubMed, Embase, Cochrane Library and CINAHL (January 2023). The search included search terms and synonyms for ‘hepatic impairment’, ‘ceftriaxone’, ‘meropenem’, ‘vancomycin’, ‘gentamicin’, ‘nitrofurantoin’, ‘fosfomycin’, ‘piperacillin-tazobactam’, ‘cirrhosis’ and ‘pharmacokinetics’ and can be found in Tables S1–S4 (available as Supplementary data at *JAC* Online). Two investigators (H.C. and M.L.) independently screened the titles and abstracts using Rayyan, a web application designed for the process of screening for systematic reviews with the help of automation tools.^18^ Studies reporting PK parameters of the antibiotics of interest were selected for full-text review. Conflicts were discussed with a third researcher (R.W.) until consensus was reached. Literature lists of included studies were examined for additional available studies.

### Eligibility criteria and data extraction

Studies were included if they met all of the following criteria: (i) study population (partially) contains adults (≥18 years) diagnosed with cirrhosis; (ii) patients were treated with ≥1 antibiotic of interest; and (iii) reporting ≥1 PK parameter of interest based on blood or ascites sampling. Studies were excluded if any of the following criteria were met: (i) review or conference abstract; (ii) *in vitro*/animal study; or (iii) not published in English or Dutch language.

The following PK parameters of interest were defined: AUC (mg·h/L), CL, renal clearance (CL_r_) and non-renal clearance (CL_nr_) (L/h), *t*_½_ (h), peak concentration (*C*_max_) (mg/L), trough concentration (*C*_min_) (mg/L) and volume of distribution (*V*_d_) (L). We furthermore looked for PTA, area under the unbound concentration–time curve over the MIC (*f*AUC/MIC) for fosfomycin and vancomycin, peak of plasma concentration over MIC (*C*_max_/MIC) for gentamicin, percentage of a dosing interval that unbound plasma concentrations remain above the MIC (%*fT*_>MIC_) for ceftriaxone, meropenem, piperacillin/tazobactam and nitrofurantoin.^19^ In addition, the following data were extracted from included studies: author, study design, year of publication, country, number of patients, patient population, age, bodyweight, intervention, sample type, dose and frequency. Definitions of ‘liver cirrhosis’ and ‘sepsis/septic shock’ were left at the discretion of the authors of the individual studies.

Data were converted to similar units of measurement, if necessary. In case of incomplete data or unavailable full text (abstract or poster only) the corresponding authors were contacted to retrieve additional data.

### Methodological quality

The quality of selected studies was assessed with ClinPK, a quality assessment tool designed for clinical PK studies.^20^ This 24-item statement checklist is a commonly used quality assessment tool for systematic reviews concerning the PK of antibiotics and other medications.^21–23^ See Table S5 for a detailed description of the ClinPK quality assessment tool.

### Statistical analysis

Continuous data were reported as mean with range, standard deviation or IQR. PK data from included studies were divided into three categories: patients with cirrhosis; patients with cirrhosis and overt ascites as a sign of clinically relevant portal hypertension; and patients with cirrhosis and impaired renal function as defined by included studies. The defined PK parameters of interest were used to determine whether the clinical state of cirrhosis, cirrhosis with clinically relevant portal hypertension or cirrhosis in combination with AKI affect the PK of the antibiotics of interest.

## Results

### Search results

**Figure 1.**
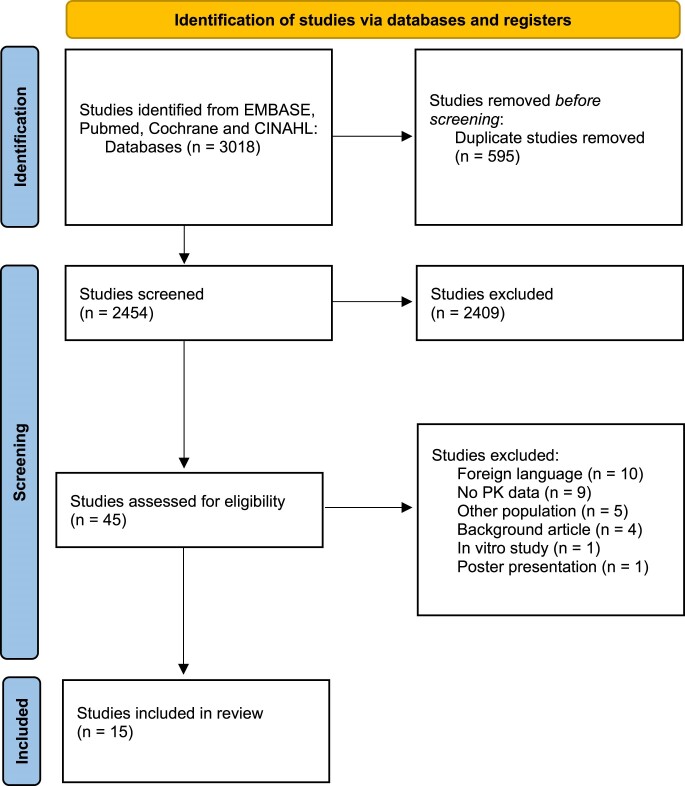
Flowchart of study selection process. Source: Page *et al.*^24^ This figure appears in colour in the online version of *JAC* and in black and white in the print version of *JAC*.

The search delivered 3018 results (Figure 1). The title and abstract of 2454 articles were screened for eligibility. The full texts of 45 studies were screened. Finally, a total of 15 studies met the predefined eligibility criteria. Included studies reported PK data on ceftriaxone (*n* = 5), gentamicin (*n* = 3), meropenem (*n* = 5) or vancomycin (*n* = 2) (Table 1). We did not identify eligible studies for fosfomycin and nitrofurantoin. We did identify three studies on piperacillin/tazobactam; however, as all these studies are incorporated in the dosing advice reported on the website ‘Drugs in liver cirrhosis’, we excluded all three studies.^17^

**Table 1.** Study characteristics of included studies

Study	Year	Design	Country	Participants	*N* (male/female)	Number with sepsis/septic shock	Age (years)	Weight (kg)	Sample	PK parameters	ClinPK score
Ceftriaxone											
Gómez-Jiménez^25^	1993	Prospective	Spain	Cirrhosis and SBP	30 (26/4)	NR, 2 died from septic shock	58.6 ± 10.7	NR	Ascitic fluid, serum	*C*_min_, *C*_max_	10/20
Hary^26^	1989	Prospective	France	Child–Pugh B or C	8 (4/4)	None	60 ± 14	75 ± 15	Ascitic fluid, serum	AUC, CL, *t*_½_, *V*_d_	11/20
Joos^27^	1984	Prospective	Switzerland	Liver disease and severe renal failure with severe infections	6 (NR)	NR	54 (3.1–81.6)	NR	Serum	*t*_½_	12/21
Schleibinger^28^	2015	Prospective	Germany	ICU patients with liver cirrhosis	2 (1/1)	1 (of 2)	60 and 61	60 and 80	Serum, serum (unbound drug)	CL, *t*_½_, *V*_d_	15/20
Stoeckel^29^	1984	Prospective	Switzerland	Cirrhosis with and without ascites	10 (6/4)	NR	43.1 (31–57)	43–89	Serum, serum (unbound drug)	CL, *t*_½_, *V*_d_	14/20
Gentamicin											
Berger^30^	1984	Prospective	Israel	Cirrhosis and SBP	30 (NR)	7 (of 30)	52.7 (24–74)	NR	Ascitic fluid, serum	*C*_min_, *C*_max_	3/21
Fevery^31^	1983	Prospective	Belgium	Cirrhosis and ascites	8	None	34–75	NR	Ascitic fluid, serum	*t*_½_	6/20
Richey^32^	1981	Prospective	USA	Cirrhosis and SBP	12 (NR)	NR	NR	NR	Ascitic fluid, serum	*C*_max_	12/20
Meropenem											
Bastida^33^	2020	Prospective	Spain	Decompensated cirrhosis and severe infections	54 (43/11)	23 (of 54) with septic shock	60 ± 11	BMI 29 ± 6	Serum	CL, *V*_d_	15/21
Grensemann^34^	2020	Prospective	Germany	ACLF and renal failure with CVVHD	8 (6/2)	5 (of 8) with sepsis/septic shock	59 (46–68)	78 (60–81)	Serum	CL, *t*_½_, *V*_d_,	18/22
Griemsmann^35^	2022	Prospective	Germany	Decompensated cirrhosis and nSBP	7 (4/3)	NR	51 [20]	67 [19]	Ascitic fluid, serum	AUC, *C*_max_, *C*_min_	10/19
Lheureux^36^	2015	Retrospective	Belgium	Critically ill patients with cirrhosis	22 (NR)	NR, 45% of all study participants (including those without meropenem treatment) had sepsis (*n* = 38)	56 (46–59)	70 (60–81)	Serum	*C*_max_, *C*_min_, CL, *t*_½_, *V*_d_,	15/20
Thyrum^37^	1997	Retrospective	USA	Compensated alcoholic cirrhosis	8 (7/1)	None	18–65	NR	Serum	AUC, *C*_max_, CL, *t*_½_, *V*_d_	16/20
Vancomycin											
Brunetti^38^	2020	Retrospective	South Korea	Child–Pugh B and C	171 (112/59)	NR	59.3 ± 15.3	BMI 29.6 ± 9.9	Serum	AUC, *C*_min_, CL, *t*_½_, *V*_d_	13/20
Harada^39^	1999	Prospective	Japan	Liver disease including cirrhosis	3 (NR)	None	68.3 ± 4.6	55.4 ± 4.9	500 mg IV×** **1 dose	CL, *t*_½_	12/20

CVVHD, continuous venovenous haemodialysis; nSBP, nosocomial SBP; NR, not reported.

### Quality assessment

We used ClinPK for quality assessment of included studies. Eligibility criteria of study participants were only described in 8 out of 15 included studies. Registration of coadministered drugs (or lack thereof) was only described by three. Seven out of 15 studies were compliant with 60% to 79% of the checklist items of the ClinPK assessment tool. Six studies were compliant with less than 60% of the checklist items and only two studies were compliant with at least 80% of the checklist items, indicating that only a small minority of studies (2 out of 15) were deemed high quality. A detailed overview of the quality assessment of individual studies is described in Table S6.

### Ceftriaxone

Two clinical studies compared blood PK parameters of ceftriaxone in cirrhotic patients with healthy controls. Study participants received a single dose of 1 g IV ceftriaxone (Table 2).^26,29^ One of these studies compared eight patients with liver cirrhosis and ascites, classified as Child–Pugh B or C, with eight healthy controls. Patients with cirrhosis tended to exhibit higher total CL_r_ and CL_nr_ compared with controls, resulting in a decrease in total AUC.^26^ The authors did not stratify for severity of cirrhosis, e.g. by applying the established Child–Pugh classification. In the second study, a decrease in CL_nr_ of unbound drug concentrations was observed, which corresponded with the degree of liver disease severity (Child–Pugh classification). This study included healthy controls (*n* = 8), patients with cirrhosis (*n* = 4) and patients with cirrhosis and ascites (*n* = 6).^29^ Notably, in patients with cirrhosis and ascites, next to a decrease in unbound CL_nr_, an increase in unbound CL_r_ was observed.

**Table 2.** PK parameters of included studies

Study, year	Patients	Dose and frequency^a^	Sample type, amount and state	Mean (range), mean [IQR] or mean ± SD						
				*C*_max_ (mg/L)	*C*_min_ (mg/L)	*V*_d_ (L)	*t*_½_ (h)	CL (mL/min)	AUC (mg·h/L)	Other PK parameters
Ceftriaxone										
Gómez-Jiménez, 1993	Child–Pugh class A (*n* = 2), B (*n* = 7) or C (*n* = 21)	2 g IV** **×** **1 dose/day, multiple dose, sample after 3 doses (72 h)	Ascites	64 (31–175)	29 (4–86)	—	—	—	—	Ascites:MIC >100 Ascites:serum *C*_max_ 0.34 ± 0.21 Ascites:serum *C*_min_ 0.56 ± 0.12
			Serum	201 (110–371)	55 (8–163)	—	11.6 ± 4.4	—	—	
Hary, 1989	Child–Pugh class B (*n* = 5) or C (*n* = 3)	1 g IV** **×** **1 dose (over 20 min), single dose, multiple samples, first sample after end of infusion (0.5–48 h)	Ascites	13.6 ± 3.2	—	—	—	—	—	—
			Serum	—	—	0.23 ± 0.06 L/kg	10.69 ± 4.27	20.7 ± 9.3	956 ± 375	CL_r_ 10.2 ± 5.1 CL_nr_ 11.3 ± 5.3
	Healthy subjects (*n* = 8)	1 g IV** **×** **1 dose	Serum	—	—	0.13 ± 0.03 L/kg	9.05 ± 1.73	13.1 ± 4.8	1399 ± 350	CL_r_ 6.0 ± 3.4 CL_nr_ 7.2 ± 0.9
Stoeckel, 1984	Cirrhosis with ascites (*n* = 6)	1 g IV** **×** **1 dose	Serum	—	—	0.228 ± 0.077 L/kg	9.7 ± 1.83	0.390 ± 0.156 mL/min/kg	—	—
			Serum unbound drug	—	—	1.22 ± 0.53	8.8 ± 0.92	2.06 ± 0.85 mL/min/kg	—	—
	Cirrhosis without ascites (*n* = 4)	1 g IV** **×** **1 dose	Serum	—	—	0.109 ± 0.035 L/kg	8.0 ± 1.98	0.235 ± 0.106 mL/min/kg	—	—
			Serum unbound drug	—	—	1.24 ± 0.42	7.6 ± 1.95	2.70 ± 0.93 mL/min/kg	—	—
	Healthy subjects (*n* = 8)	1 g IV** **×** **1 dose	Serum	—	—	0.142 ± 0.017 L/kg	8.4 ± 1.82	0.226 ± 0.064 mL/min/kg	—	—
			Serum unbound drug	—	—	2.93 ± 0.63	7.8 ± 1.54	4.59 ± 1.29 0.064 mL/min/kg	—	—
Ceftriaxone in dual impairment										
Joos, 1984	Concomitant liver disease and severe renal failure (*n* = 6)	1 g (*n* = 5) or 2 g (*n* = 1) IV** **×** **1 dose per day, sample between Days 3 and 11 before and, usually, after 2 h from start of infusion	Serum	—	—	—	33.6 ± 13.3	—	—	—
	Normal renal and hepatic function (*n* = 44)	0.8–3.0 g IV** **×** **1 dose per day		—	—	—	8.2 ± 2.7	—	—	—
Schleibinger, 2015	Liver cirrhosis (*n* = 1)	2 g IV** **×** **1 dose, median 3 samples, total 69, sampling time chosen opportunistically, aim of capturing 1–2 dosing intervals, 3–4 samples	Serum	—	—	20.0	14.5	15.83	—	—
			Serum unbound drug	—	—	34.1	13.2	29.83	—	—
	Liver cirrhosis and renal impairment (*n* = 1)	2 g IV** **×** **1 dose	Serum	—	—	28.0	19.5	16.67	—	—
			Serum unbound drug	—	—	31.2	15.6	23	—	—
Gentamicin										
Berger, 1984	Cirrhosis with SBP (*n* = 30)	Parenteral 240–360 mg/day, after 48 h, between <2 and >5 h	Ascites	1.66 ± 0.99	1.20 ± 0.29	—	—	—	—	*C*_ascites:serum_ 24%–217%
			Serum	2.44 ± 0.98	1.84 ± 0.98	—	—	—	—	
Fevery, 1983	Cirrhosis and ascites (*n* = 8)	80 mg IV** **×** **1 dose, single dose, before and 1–4 and 8–20 h after	Ascites	—	—	—	—	—	—	*C*_ascites:serum_ 100% after 3–5 h
			Serum	—	—	—	2.83 ± 0.75	—	—	
Richey, 1981	Cirrhosis and ascites with SBP (*n* = 9)	3–5 mg/kg/q8h, minimum 2 days before sampling, 1 or 4 h after 30 min infusion samples	Ascites	4.2 (<2.0–5.7)	—	—	—	—	—	Peritoneal penetration 67.8% (35.9%–101.2%)
		3–5 mg/kg/q8h	Serum	6.1 (4.0–8.2)	—	—	—	—	—	
Meropenem										
Thyrum, 1997	Cirrhosis (*n* = 8)	1 g IV** **×** **6 doses, single and steady-state dosages, multiple samples on Day 1 and Day 6 (described as first day and steady-state samples)	Serum	51.2 ± 3.6^b,c^	—	18.8 ± 1.4^b^ L	1.4 ± 0.1^b^	250.4 ± 20.5^b^	AUC_0–6_ 69.2 ± 4.6^b^	Significant decrease in single-dose meropenem urine excretion
	Matched controls (*n* = 8)	1 g IV** **×** **6 doses		54.6 ± 4.0^b,c^	—	22.2 ± 1.4^b^ L	1.2 ± 0.1^b^	236.6 ± 18.0^b^	AUC_0–6_ 72.6 ± 4.8^b^	
Meropenem in dual impairment										
Bastida, 2020 (popPK)	Child–Pugh A (*n* = 8), B (*n* = 16) or C (*n* = 30)	Mean daily dose 3.9 ± 1.7 g, blood samples (5 mL) were collected once steady state was achieved, Samples: 30 min bolus: before, 0 min, 15 min, 60 min, 3–8 h 4 h infusion: pre-dose, 4, 4.15, 5, 6–8 h 12 h infusion: Day 1, and 24 h later	Serum	—	—	ACLF 42.2, NLF 28.2	—	8.35 L/h	—	CL_CR_ significantly associated with CL_meropenem_ PTA was used as a cut-off point to assess their covariables.
Grensemann, 2020 (popPK)	ACLF and renal failure (*n* = 8)	1 g IV q8h, steady state, 0 h first infusion, 1, 2, 4, 6, 8 h, after start of infusion. T8 was before next infusion, also 24 and 48 h samples Monte Carlo simulations: A: 1 g three times a day SI B: 2 g loading plus 1 g prolonged infusion three times a day C: 2 g three times a day SI D: 2 g loading and continuous	Serum	—	—	35.5	9.0	5.06 L/h	—	PTA for Enterobacterales was achieved 100% in all dosing regimens. PTA for *Pseudomonas* spp.: Day 1/7 A: 18%/80% B: 94%/88% C: 85%/98% D: 100%/100%
	NLF and renal failure (*n* = 11)		Serum	—	—	18.5	5.0	5.11 L/h	—	PTA for Enterobacterales was achieved 100% in all dosing regimens. Day 1/7 A: 48%/65% B: 91%/83% C: 91%/93% D: 100%/100%
Griemsmann, 2022	Decompensated cirrhosis with SBP (*n* = 7)	Median 3 g, 0.5 g/day, steady state, Day 1 sample, and on Days 3 to 5 multiple at 0 to 960 min after infusion, remaining treatment days, 1 sample before infusion	Ascites	26.0 [5.1]	12.2	—	—	—	AUC_0–8_ single dose 124 [41] AUC_0–8_ multiple dose 125 [56]	*t*_4×MIC_: 0.5 h *t_C_*_max_: 0.5 h
			Serum	44.7 [3.6]	12.4	—	—	—	AUC_0–8_ single dose: 178 [73] AUC_0–8_ multiple dose: 174 [77]	*t_C_*_max_: 2 h *t*_>4×MIC_: median 100%
Lheureux, 2016	Cirrhosis (*n* = 22)	Median total daily dose 3 (2–3) g, two samples, one before and one 2 h after infusion assuming steady state was reached	Serum	24.1 [19.2–27.5]	7.7 [4.0–12.4]	0.43 [0.37–0.80]	4.9 [3.4–5.9]	96 [59–142]	—	—
	Matched group (*n* = 22)	Median total daily dose 3 (2–3) g	Serum	19.4 [9.9–27.4]	5.4 [2.5–11.9]	0.77 [0.47–1.12]	4.3 [3.3–6.6]	102 [60–278]	—	—
Vancomycin										
Brunetti, 2020	Moderate to severe liver disease (*n* = 171)	Mean daily dose 2768 ± 1014 mg, steady state, through to concentration 30 min prior to upcoming dose with 1 h leeway	Serum	—	17.5 ± 8.4	0.93 ± 0.12	13.4 ± 6.5	4.8 ± 2.0	AUC:MIC 549.4 ± 217.2	—
	No to mild liver disease (*n* = 237)	Mean daily dose 3030 ± 969 mg, multiple samples 0, 1, 2, 3, 5, 7, 12 ,24 ,48 h	Serum	—	15.3 ± 5.2	0.94 ± 0.13	11.3 ± 5.1	5.8 ± 2.4	AUC:MIC 497.5 ± 117.3	—
Harada, 1999	Liver disease including cirrhosis (*n* = 7)	500 mg IV** **×** **1 dose	Serum	—	—	—	7.5 ± 0.7	1.66 ± 0.95	—	—

*C*_ascites:serum_, ratio between ascites and serum concentration; NLF, no liver failure; *t*_4×MIC_, time to 4×MIC concentration; *t_C_*_ascites > _*_C_*_serum_, time to *C*_ascites _> *C*_serum_; *t_C_*_max_, time to *C*_max_; *V*_area_, *V*_d_ based on AUC; *V*_dss_, *V*_d_ at steady state.

^a^Only intermittent administration was evaluated in included studies.

^b^Study used SE instead of SD.

^c^Only first dose PK data were reported.

Two studies reported on ceftriaxone ascites PK parameters.^25,26^ Both showed that ceftriaxone rapidly enters the ascitic fluid compartment, with one study reporting a ascites:blood *C*_max_ ratio of 0.34.

Two studies reported ceftriaxone PK data in cirrhotic patients with impaired renal function.^27,28^ In one of them, conducted in ICU patients, two patients had undefined liver function impairment, with one having concomitant impaired renal function.^28^ A single IV dose of 2 g ceftriaxone was administered and a relatively higher unbound ceftriaxone CL of 1.79 was found in the patient with impaired liver function but intact renal function when compared with the patient who had accompanying impaired renal function (1.38 L/h). The second study did not provide data on AUC or CL but did report a 4-fold increase in total *t*_½_ (33.6 versus 8.2 h) in six patients with dual impairment (liver and kidney) who received a single IV dose of 1 g ceftriaxone.^27^

### Gentamicin

All three included studies compared blood and ascitic fluid samples in patients with cirrhosis.^30–32^ In one study, IV gentamicin at a dose of 240–360 mg/day was administered to 30 patients with spontaneous bacterial peritonitis (SBP). The gentamicin penetration rate (ascites/blood ratio) ranged from 24% to 217% and the serum *C*_max_ ranged from 3.9 to 6.75 mg/L.^30^ Another study included nine patients with SBP treated with IV gentamicin at a dose of 3–5 mg/kg at 8 h intervals and reported a mean ascites penetration ratio of 67.8% (IQR 35.9%–101.2%).^32^ The third study administered a single 800 mg IV gentamicin dose to 10 patients without an active bacterial infection. The study observed increasing ascites concentrations from 1 to 4 h after injection and remarkably a subsequent equalization of ascites and blood concentrations between 3 and 5 h post-injection.^31^ It was estimated that at least 50% of patients had decreased renal function prior to gentamicin administration. However, the study did not report on the degree of renal function impairment, nor did it report follow-up measurements of renal function after administration of gentamicin. No further PK parameters were described in these studies.

### Meropenem

One study reported on clinical PK data in 16 patients with compensated cirrhosis and intact renal function.^37^ Compared with matched healthy controls, no difference in AUC or CL between the groups was observed after the first dose and in steady state.

Four studies described patients with both impaired liver and renal function.^33–36^ The first study compared 8 patients with acute-on-chronic liver failure (ACLF) with 11 patients without liver failure and did not observe a difference in meropenem CL between the groups.^34^ Adequate PTA for Enterobacterales was achieved for all dosing regimens, but 100% PTA for *Pseudomonas* spp. was only achieved in patients receiving a continuous infusion of meropenem 3 g/day after a short-term infusion loading dose of 2 g. The second study was a matched case–control study comparing blood β-lactam concentrations in 22 critically ill patients, partially treated with renal replacement therapy. This study found no difference in meropenem CL between patients with cirrhosis and matched controls.^36^

The third study described ascites samples in seven patients with decompensated cirrhosis and impaired renal function with SBP and found a 1.5 times higher AUC_0–8_ in blood compared with ascites.^35^

The fourth study was a population PK study in 54 patients with severe infections and varying Child–Pugh classifications. The study identified creatinine CL (CL_CR_) as a significant covariate next to model for end-stage liver disease (MELD) score and presence of ACLF, influencing the blood meropenem concentration variability, leading to the lowest meropenem CL in patients with the lowest CL_CR_ and the highest MELD score.^33^ A higher MELD score was associated with an increased PTA.

### Vancomycin

Two studies reported vancomycin PK data in patients with cirrhosis.^38,39^ One study compared the administration of 3 g/day vancomycin in 171 patients with moderate (Child–Pugh B) to severe (Child–Pugh C) liver disease with 237 patients with no to mild (Child–Pugh A) liver disease and found a 20% decrease in vancomycin CL (2.88 versus 3.48 L/h) and a 10% increase in mean AUC:MIC (549.4 versus 497.5 mg·h/L) in patients with Child–Pugh B–C cirrhosis.^38^ The other study presented the PK of vancomycin in preoperative patients.^39^ A higher CL_nr_/CL_total_ (0.51 versus 0.19) was found in three patients with liver disease compared with patients with malignancy.^39^ A negative correlation was found between vancomycin CL and protein binding percentage.

## Discussion

This systematic review was conducted to evaluate the extent and quality of available literature concerning PK alterations of antibiotics in patients with cirrhosis for which currently no evidence-based dosing recommendations exist in public Dutch resources. We showed that the available PK studies of selected antibiotics are limited and of moderate quality and, mostly, with a low number of included patients, increasing the risk of coincidental findings. Importantly, few studies stratified patients according to the severity of cirrhosis to evaluate the effect of increasing liver disease severity on PK and the potential complex interaction with concomitant AKI. In addition, studies focusing on PK/ pharmacodynamic (PK/PD) target attainment, such as attaining enough time above MIC for ceftriaxone and meropenem or sufficiently high AUC/MIC for vancomycin, were not found. As we did identify studies describing alterations in AUC and CL that could require consideration when dosing antibiotics in patients with liver cirrhosis, further research is essential to provide specific dosing recommendations.

In 2014, a systematic review was performed that focused on PK alterations of antibiotics in patients with cirrhosis to provide dosing recommendations and ultimately aimed to develop a decision algorithm.^14^ Similar to the current review, ceftriaxone and nitrofurantoin were studied. Despite widening our literature search by also assessing the potential impact of AKI next to cirrhosis on PK parameters and exploring the effect of concomitant presence of ascites (in the context of portal hypertension) on PK parameters, our search did not retrieve additional studies.

For ceftriaxone, the included studies suggest that total CL is increased in patients with cirrhosis when compared with healthy controls (0.78–1.8 versus 0.58 to 1.45 L/h).^40^ This finding could be explained by the high rate (90%–95%) of protein binding of ceftriaxone; liver cirrhosis leads to impairment of albumin synthesis (the most abundant protein in the human body), which in turn decreases the possibility of ceftriaxone binding to protein. Given that ceftriaxone has saturable protein binding and follows first-order elimination kinetics, the extra unbound ceftriaxone amount is expected to be immediately distributed and eliminated, leading to unchanged unbound concentrations, lower total concentrations and thus a lower bound fraction and increased total CL.^41–43^ Since the unbound concentration is, from a theoretical perspective, the microbiologically and pharmacologically active moiety, the observed increase in total ceftriaxone CL may be without clinical significance, especially if no changes in unbound ceftriaxone exposure are observed. However, the effect on unbound CL was investigated in only one study and showed a decrease in CL_nr_ relative to healthy controls.^29^ Based on available data it remains unclear whether these changes could lead to accumulation of unbound ceftriaxone and increase the risk of drug toxicity.

Both included studies that reported ascitic fluid PK data on ceftriaxone suggest rapid penetration of ceftriaxone through the peritoneal membrane,^25,26^ with a strong association between concentrations of ceftriaxone in blood and peritoneal fluid (i.e. ascites).^44^ In the context of treating SBP, rapid penetration into the peritoneal cavity (the target site of infection) is beneficial when trying to achieve unbound concentrations above the MIC for causative Gram-negative enteric bacteria.^45^ These outcomes suggest that ceftriaxone is a suitable option for SBP treatment. This is also supported by the fact that current guidelines recommend a third-generation cephalosporin for the treatment of community-acquired SBP.^13^ Unfortunately, this study did not report data on CL, AUC or %*fT*_>MIC_. None of the included studies regarding ceftriaxone were deemed to be high quality. The limited number of included patients in the two available studies prevent definitive conclusions being drawn on the dosing recommendation for ceftriaxone in patients with liver cirrhosis and the observations made in these studies do warrant further investigation.

All three included gentamicin studies were conducted in the 1980s and only reported ascitic fluid PK data. These described slow diffusion of gentamicin into ascites based on ascites concentration levels. Two of three studies were performed in patients with SBP.^30,32^ Currently, European guidelines advise against the use of aminoglycosides in cirrhosis as empirical treatment for SBP due to their high risk of nephrotoxicity in cirrhosis, which hampers the initiation of new well-designed studies on their safety for its use in treating infections in patients with cirrhosis.^13,46^ Nevertheless, future studies should focus on the PK parameters and safety of aminoglycosides in patients with cirrhosis and a severe infection resistant to other antibiotic treatments and should determine if certain subgroups of patients with relative mild liver insufficiency and intact renal function can effectively and safely receive aminoglycoside treatment.

The included meropenem studies with cirrhotic patients with intact renal function showed no significant differences in the PK parameters compared with healthy controls, which supports the hypothesis of a negligible effect of the presence of cirrhosis on plasma meropenem PK parameters. Approximately 70% of meropenem is renally excreted, which could explain the decrease in CL in the presence of AKI, as was found in one study including patients with dual impairment.^33,47,48^ This is further supported by the other study in which 8 patients with both liver cirrhosis and AKI were compared with 11 patients with AKI and intact liver function, which did not show a difference in CL between both groups.^34^ However, these results are confounded because study participants received renal replacement therapy.^49^ In contrast to these studies, one population PK study suggests that both cirrhosis and AKI do have an independent effect on meropenem CL as both were included as covariates after multivariate analysis.^33^ Furthermore, one included study reported a 2-fold increase in *V*_d_ in ACLF patients, possibly due to the presence of ascites in this patient group.^34^ This increased *V*_d_ because of the presence of ascites could indicate the need for a loading dose when starting treatment with meropenem in patients with cirrhosis and ascites.^50^

While vancomycin is almost exclusively renally excreted in unchanged form and liver metabolism is minimal,^51,52^ one study assessing vancomycin toxicity observed a statistically significant decrease in CL and an increase in AUC in 171 patients with moderate to severe liver dysfunction.^38^ Renal function is difficult to estimate in patients with liver disease because of malnutrition and decreased muscle mass.^53^ This leads to an overestimation of true glomerular filtration rate (GFR), especially in patients with ascites.^12^ The results of this study could suggest lower initial dosing following a loading dose and to intensify blood level monitoring (preferably within 24 h) for patients with severe liver disease with apparent ‘normal estimated GFR’ to prevent possible vancomycin-related nephrotoxicity. Alternatively, 24 h urine collection could be used to assess renal function for dosage adjustments.

In this study, we systematically evaluated the extent and quality of available literature concerning PK alterations of antibiotics in patients with liver cirrhosis, patients with cirrhosis and overt ascites, and patients with liver cirrhosis and impaired renal function. While the limited available data precluded specific dosing recommendations, available data suggest relevant PK alterations in patients with cirrhosis, which need to be investigated by future well-designed prospective clinical PK studies.

This systematic review has several limitations. First, we limited our search to only a subset of antibiotic drugs. This was based on the fact that these antibiotics had no specific dose recommendations available at the time of study conception and were deemed clinically relevant. It is possible that other relevant antibiotics, not common in Dutch daily practice, also have no dose recommendations and were not included. Another limitation is the heterogeneity of the included study participants (patients who underwent continuous haemodialysis were included, some patients had SBP and others were admitted to the ICU). This heterogeneity prevents a formal comparison of PK parameters between study populations. The quality assessment revealed that many studies scored moderately on the methodology section, which was primarily related to the use of outdated methodology or limited description of applied methodology in available studies.

Finally, stratification for the severity of cirrhosis (e.g. by Child–Pugh classification) was rarely implemented and separately reported for each Child–Pugh class. Only 4 out of the 15 studies reported baseline data on liver disease severity of study participants and only one included study reported PK data stratified per Child–Pugh class. Absence of these data prevents the development of specific dosing recommendations.

This study revealed important knowledge gaps. Future studies should examine possible PK alterations of nitrofurantoin, fosfomycin and piperacillin/tazobactam in patients with cirrhosis. Ceftriaxone studies should focus on unbound ceftriaxone measurements and examine whether excessive accumulation develops in blood or ascites, especially in the case of dual impairment, and whether this leads to drug-induced toxicity. The latter also goes for meropenem studies and even more so for vancomycin studies. Also, in general, future studies should especially focus on the combined effect of liver cirrhosis, portal hypertension and AKI on individual PK parameters and PK/PD target attainment. To compare study findings and specifically enable dosing recommendations for patients with cirrhosis, uniform definitions for liver disease severity should be used to stratify patients, for example Child–Pugh score, MELD scores (MELD-Na, MELD 3.0) and/or albumin-bilirubin (ALBI) score.

In conclusion, this systematic review showed that the number of available PK studies to assess the extent of PK alterations of ceftriaxone, fosfomycin, gentamicin, meropenem, nitrofurantoin, piperacillin/tazobactam and vancomycin in adult patients with cirrhosis is limited and of moderate quality. Also, the role of concomitant AKI remains to be elucidated. Future studies should assess the true impact of cirrhosis and AKI on PK parameters and PK/PD target attainment of antibiotics in patients with cirrhosis to facilitate specific dosing recommendations.

## Supplementary Material

dkae310_Supplementary_Data

## Supplementary data

Tables S1 to S6 are available as Supplementary data at *JAC* Online.
