# Supplementary material for: Pharmacokinetics of ceftriaxone, gentamicin, meropenem and vancomycin in liver cirrhosis: a systematic review
Source: J Antimicrob Chemother. 2024 Sep 18;79(11):2750–61. doi: 10.1093/jac/dkae310 (PMC11531807; doi:10.1093/jac/dkae310)
Supplement: dkae310_Supplementary_Data [file dkae310_supplementary_data.docx]

**SUPPLEMENTARY DATA**

**Table S1**. Pubmed strategy

| 1101 results  ("Liver Cirrhosis"[Mesh] OR cirrho*[tiab] OR hepatic impairment[tiab] OR liver impairment[tiab] OR hepatic dysfunction[tiab] OR liver dysfunction[tiab] OR hepatic insufficiency[tiab] OR liver insufficiency[tiab])  AND  ("Anti-Infective Agents/pharmacokinetics"[Mesh] OR "Anti-Infective Agents/therapeutic use"[Mesh] OR "Anti-Bacterial Agents" [Pharmacological Action] OR "Piperacillin"[Mesh] OR "Tazobactam"[Mesh] OR "Piperacillin, Tazobactam Drug Combination"[Mesh] OR "Meropenem"[Mesh] OR "meropenem and vaborbactam" [Supplementary Concept] OR "Vancomycin"[Mesh] OR "Nitrofurantoin"[Mesh] OR "Fosfomycin"[Mesh] OR "Gentamicins"[Mesh] OR "Tobramycin"[Mesh] OR "Ceftriaxone"[Mesh] OR piperacillin[tiab] OR tazobactam[tiab] OR meropenem[tiab] OR vancomycin[tiab] OR nitrofurantoin[tiab] OR fosfomycin[tiab] OR gentamicins[tiab] OR tobramycin[tiab] OR ceftriaxone[tiab])  AND  ("Pharmacokinetics"[Mesh] OR "pharmacokinetics" [Subheading] OR "Liver Function Tests"[Mesh] OR pharmacokinetic*[tiab] OR pharmaco-kinetic*[tiab] OR pharmacological kinetic*[tiab] OR kinetic profile[tiab] OR pharmacodynamic*[tiab] OR toxicokinetic*[tiab] OR tissue distribution[tiab] OR PK parameter*[tiab] OR AUC[tiab] OR Cmax[tiab] OR tmax[tiab])  NOT  (("Animals"[Mesh] OR "Models, Animal"[Mesh] OR "Animal Experimentation"[Mesh] OR rodent∗[ti] OR rabbit∗[ti] OR mice[ti] OR mouse[ti] OR murine[ti] OR rat[ti] OR rats[ti] OR murine[ti]) NOT "Humans"[Mesh])  NOT  ("Pediatrics"[Mesh] OR child*[ti] OR pediatric*[ti] OR paediatric*[ti]) |
| --- |

**Table S2.** Cochrane strategy

| ID Search Hits  #1 (cirrho* or hepatic impairment or liver impairment or hepatic dysfunction or liver dysfunction or hepatic insufficiency or liver insufficiency):ti,ab,kw 17325  #2 (piperacillin or tazobactam or meropenem or vancomycin or nitrofurantoin or fosfomycin or gentamicins or tobramycin or ceftriaxone):ti,ab,kw 8233  #3 MeSH descriptor: [Anti-Infective Agents] explode all trees and with qualifier(s): [pharmacokinetics - PK] 2725  #4 #2 or #3 10750  #5 (pharmacokinetic* or pharmaco-kinetic* or pharmacological kinetic* or kinetic profile or pharmacodynamic* or toxicokinetic* or tissue distribution or PK parameter* or AUC or Cmax or tmax OR liver function test*):ti,ab,kw 117538  #6 #1 AND #4 and #5 in Trials 60 |
| --- |

**Table S3.** CINAHL search strategy

| 107 results  (MH "Liver Cirrhosis+") OR TI ( cirrho* or hepatic impairment or liver impairment or hepatic dysfunction or liver dysfunction or hepatic insufficiency or liver insufficiency) OR AB ( cirrho* or hepatic impairment or liver impairment or hepatic dysfunction or liver dysfunction or hepatic insufficiency or liver insufficiency)  AND  (MH "Antiinfective Agents+/PK") OR (MH "Ceftriaxone") OR TI ( Piperacillin or Tazobactam or Meropenem or Vancomycin or Nitrofurantoin or Fosfomycin or Gentamicins or Tobramycin or ceftriaxone) OR AB ( Piperacillin or Tazobactam or Meropenem or Vancomycin or Nitrofurantoin or Fosfomycin or Gentamicins or Tobramycin or ceftriaxone)  AND  (MH "Pharmacokinetics+") OR (MH "Liver Function Tests") OR TI ( pharmacokinetic* or pharmaco-kinetic* or pharmacological kinetic* or kinetic profile or pharmacodynamic* or toxicokinetic* or tissue distribution or PK parameter* or AUC or Cmax or tmax ) OR AB ( pharmacokinetic* or pharmaco-kinetic* or pharmacological kinetic* or kinetic profile or pharmacodynamic* or toxicokinetic* or tissue distribution or PK parameter* or AUC or Cmax or tmax )  NOT  (MH "Pediatrics+") OR TI ( child* or pediatric* or paediatric* ) |
| --- |

**Table S4.** EMBASE search strategy

| **#** | **Searches** | **Results** |
| --- | --- | --- |
| 1 | exp liver cirrhosis/ | 208854 |
| 2 | (cirrho* or hepatic impairment or liver impairment or hepatic dysfunction or liver dysfunction or hepatic insufficiency or liver insufficiency).ti,ab,kf. | 222214 |
| 3 | 1 or 2 | 275025 |
| 4 | exp antiinfective agent/pk or ceftriaxone/ or exp piperacillin plus tazobactam/ or exp piperacillin/ or exp tazobactam/ or exp piperacillin plus tazobactam/ or exp ceftolozane plus tazobactam/ or exp meropenem plus vaborbactam/ or exp meropenem/ or exp nitrofurantoin/ or exp fosfomycin plus tobramycin/ or exp fosfomycin/ or exp gentamicin/ or exp dexamethasone plus tobramycin/ or exp tobramycin/ | 343345 |
| 5 | (piperacillin or tazobactam or meropenem or vancomycin or nitrofurantoin or fosfomycin or gentamicins or tobramycin or ceftriaxone).ti,ab,kf. | 106252 |
| 6 | 4 or 5 | 379462 |
| 7 | pharmacokinetics/ or pharmacokinetics.fs. or liver function test/ | 729861 |
| 8 | (pharmacokinetic* or pharmaco-kinetic* or pharmacological kinetic* or kinetic profile or pharmacodynamic* or toxicokinetic* or tissue distribution or PK parameter* or AUC or Cmax or tmax).ti,ab,kf. | 469010 |
| 9 | 7 or 8 | 970653 |
| 10 | 3 and 6 and 9 | 1846 |
| 11 | (exp animal/ or exp animal experiment/ or exp animal model/ or exp veterinary medicine/ or (rodent* or rabbit* or rat or rats or mice or mouse or murine).ti.) not human/ | 6476410 |
| 12 | 10 not 11 | 1801 |
| 13 | exp pediatrics/ or (child* or pediatric* or paediatric*).ti. | 1440795 |
| 14 | 12 not 13 | 1749 |

**Table S5.** ClinPK quality assessment tool 24-item checklist


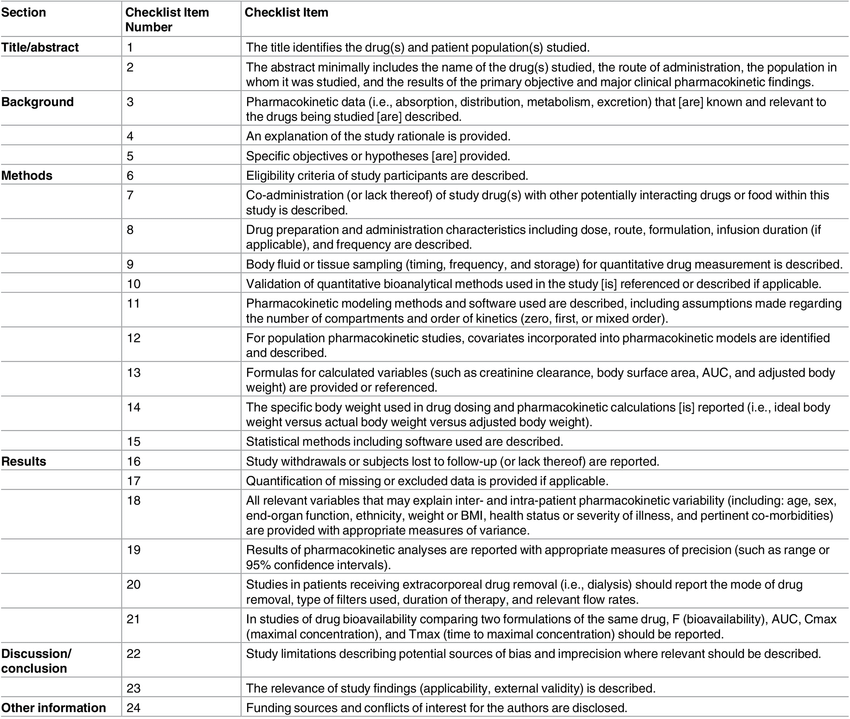


**Table S6. ClinPK** quality assessment of all included studies

| No. | 1 | 2 | 3 | 4 | 5 | 6 | 7 | 8 | 9 | 10 | 11 | 12 | 13 | 14 | 15 | 16 | 17 |
| --- | --- | --- | --- | --- | --- | --- | --- | --- | --- | --- | --- | --- | --- | --- | --- | --- | --- |
| Author, year | Bastida, 2020 | Berger, 1984 | Bourget, 1996 | Brunetti, 2020 | Fevery, 1983 | Gomez-Jimenez, 1993 | Grensemann, 2020 | Griemsmann, 2022 | Harada, 1999 | Hary, 1989 | Hary, 1991 | Joos, 1984 | Lheureux, 2016 | Richey, 1981 | Schleibinger, 2015 | Stoeckel, 1984 | Thyrum, 1997 |
| Score | 15/21 (71%) | 3/21 (14%) | 14/20 (70%) | 13/20 (65%) | 6/20 (30%) | 10/20 (50%) | 18/22 (81%) | 10/19 (52%) | 12/20 (60%) | 11/20 (55%) | 13/20 (65%) | 12/21 (57%) | 15/20 (75%) | 12/20 (60%) | 15/20 (75%) | 14/20 (70%) | 16/20 (80%) |
| 1. Title | Yes | Yes | Yes | Yes | No | Yes | Yes | Yes | Yes | Yes | Yes | Yes | Yes | Yes | Yes | Yes | Yes |
| 2. Abstract | No | No | Yes | No | Yes | No | Yes | NA | Yes | Yes | Yes | Yes | No | Yes | Yes | Yes | Yes |
| 3. PK background | Yes | No | Yes | Yes | No | Yes | Yes | Yes | Yes | No | Yes | Yes | Yes | No | Yes | Yes | Yes |
| 4. Study rationale | Yes | No | Yes | Yes | Yes | Yes | Yes | Yes | Yes | Yes | Yes | Yes | Yes | Yes | Yes | Yes | Yes |
| 5. Specific objective or hypothesis | Yes | No | Yes | Yes | No | Yes | Yes | Yes | Yes | Yes | Yes | Yes | Yes | Yes | Yes | Yes | Yes |
| 6. Eligibility criteria of participants | Yes | No | No | Yes | No | No | Yes | Yes | No | No | No | Yes | Yes | No | Yes | No | Yes |
| 7. Coadministration of drugs | No | No | Yes | No | No | Yes | No | No | No | No | No | No | No | No | No | No | Yes |
| 8. Drug preparation and administration | No | No | Yes | No | No | Yes | Yes | No | No | No | Yes | No | No | No | No | Yes | Yes |
| 9. Sampling described | Yes | No | Yes | No | Yes | Yes | Yes | Yes | Yes | Yes | Yes | Yes | No | Yes | Yes | Yes | Yes |
| 10. Validation of bioanalytical methods | Yes | No | Yes | No | No | No | Yes | No | Yes | Yes | Yes | Yes | Yes | Yes | Yes | Yes | Yes |
| 11. PK modeling described | Yes | No | Yes | Yes | No | No | Yes | No | Yes | Yes | Yes | Yes | Yes | Yes | Yes | Yes | Yes |
| 12. Population PK model covariates described | Yes | NA | NA | NA | NA | NA | Yes | NA | NA | NA | NA | NA | NA | NA | NA | NA | NA |
| 13. Formulas provided | No | No | Yes | No | No | No | No | No | No | No | No | No | No | No | No | Yes | Yes |
| 14. Body weight used | No | No | No | No | No | No | No | No | No | No | No | No | Yes | No | No | No | No |
| 15. Statistical methods described | Yes | No | Yes | Yes | Yes | Yes | Yes | No | Yes | Yes | Yes | Yes | Yes | No | Yes | Yes | Yes |
| 16. Study withdrawals reported | No | No | No | Yes | No | No | No | No | No | No | No | No | NA | No | No | No | Yes |
| 17. Missing data quantified | NA | No | NA | NA | NA | NA | NA | NA | NA | NA | NA | No | NA | NA | NA | NA | NA |
| 18. Other explanatory variables provided | Yes | No | No | Yes | No | No | Yes | Yes | Yes | Yes | Yes | No | Yes | No | Yes | Yes | No |
| 19. Results reported with precision | Yes | Yes | Yes | Yes | Yes | Yes | Yes | Yes | Yes | Yes | Yes | Yes | Yes | Yes | Yes | Yes | Yes |
| 20. Dialysis methods described | NA | NA | NA | NA | NA | NA | Yes | NA | NA | NA | NA | NA | Yes | NA | NA | NA | NA |
| 21. Bioavailability | NA | NA | NA | NA | NA | NA | NA | NA | NA | NA | NA | NA | NA | NA | NA | NA | NA |
| 22. Limitations described | Yes | No | No | Yes | No | No | Yes | No | No | No | No | No | Yes | No | Yes | No | No |
| 23. Relevance of findings described | Yes | Yes | Yes | Yes | Yes | Yes | Yes | Yes | Yes | Yes | Yes | Yes | Yes | Yes | Yes | Yes | Yes |
| 24. Funding and author COI described | Yes | No | No | Yes | No | No | Yes | Yes | No | No | No | No | Yes | Yes | Yes | No | No |

Abbreviations: COI, conflict of interest; NA, not applicable; PK, pharmacokinetic
